# Supplementary material for: On the Decoupling of Evolutionary Changes in mRNA and Protein Levels
Source: Mol Biol Evol. 2023 Jul 27;40(8):msad169. doi: 10.1093/molbev/msad169 (PMC10411491; doi:10.1093/molbev/msad169)
Supplement: msad169_Supplementary_Data [file msad169_supplementary_data.zip › mRNA_protein_evo_theory_suppmat.pdf]

On the decoupling of evolutionary changes in mRNA and protein levels: Supplementary materials

Table S1: Transcription-translation correlation and mRNA-protein correlation across replicate lineages under different combinations of mutation rates. The rate of mutations that affect transcription and the rate of mutations that affect translation were chosen such that the total mutation rate is held the same. Each mutation rate presented in the table is the expected number of mutations that affect the corresponding trait to enter the population in each time step (i.e., $2N_eU_\alpha$ or $2N_eU_\beta$, see Materials and Methods).

**Mutation rate**		**Selection regime**	**Correlation coefficient**	
Transcription	Translation		Transcription-translation	mRNA-protein
0.2	1.8	Stabilizing selection	-0.9359	0.04871
		Neutral	0.05008	0.3734
0.4	1.6	Stabilizing selection	-0.9670	-0.06934
		Neutral	0.002205	0.4444
0.6	0.4	Stabilizing selection	-0.9729	0.09305
		Neutral	-0.02003	0.5403
0.8	1.2	Stabilizing selection	-0.9761	0.1083
		Neutral	-0.0624	0.6020
1	1	Stabilizing selection	-0.9765	0.09209
		Neutral	0.0004967	0.7126
1.2	0.8	Stabilizing selection	-0.9725	0.1466
		Neutral	-0.01530	0.7618
1.4	0.6	Stabilizing selection	-0.9732	0.1604
		Neutral	-0.03154	0.8195
1.6	0.4	Stabilizing selection	-0.9640	0.1763
		Neutral	0.01579	0.9116
1.8	0.2	Stabilizing selection	-0.9273	0.3177
		Neutral	-0.05235	0.9446

Table S2: Transcription-translation correlation and mRNA-protein correlation across replicate lineages under different levels of measurement error.

**Error SD**	**Selection regime**	**Correlation coefficient**	
		Transcription-translation	mRNA-protein
0	Stabilizing selection	-0.9765	0.09209
	Neutral	0.0004967	0.7126
0.01	Stabilizing selection	-0.9670	0.05904
	Neutral	0.0003159	0.7121
0.02	Stabilizing selection	-0.9487	0.06802
	Neutral	0.003284	0.7139
0.03	Stabilizing selection	-0.9216	0.05275
	Neutral	-0.002881	0.7107
0.04	Stabilizing selection	-0.8714	0.1294
	Neutral	-0.007650	0.7072
0.05	Stabilizing selection	-0.8570	0.02024
	Neutral	-0.02017	0.7057

Table S3: Evolutionary correlations between traits under different levels of measurement error.

**Error SD**	**Selection regime**	**Correlation coefficient**	
		Transcription-translation	mRNA-protein
0	Stabilizing selection	-0.7823	0.3148
	Neutral	-0.006527	0.7025
0.01	Stabilizing selection	-0.7545	0.2266
	Neutral	-0.01005	0.7005
0.02	Stabilizing selection	-0.7302	0.1184
	Neutral	-0.02077	0.6961
0.03	Stabilizing selection	-0.7157	0.0604
	Neutral	-0.03180	0.6888
0.04	Stabilizing selection	-0.7289	-0.0004425
	Neutral	-0.05423	0.6789
0.05	Stabilizing selection	-0.7169	0.007953
	Neutral	-0.06931	0.6665

Table S4: Evolutionary correlations estimated from results of simulations along transformed trees.

**Lambda**	**Correlation coefficient**	
	Transcription-translation	mRNA-protein
1 (original)	-0.7823	0.3148
0.5	-0.9663	0.1323
0 (star tree)	-0.9760	0.1073

Table S5: Transcription-translation correlation and mRNA-protein correlation of genes in triple-gene regulatory motifs. Red numbers indicate the gene's protein level is directly subject to stabilizing selection.

**Regulatory motif**	**Correlation coefficient**					
	Transcription-translation			mRNA-protein		
	Gene 1	Gene 2	Gene 3	Gene 1	Gene 2	Gene 3
Motif 1	-0.134	-0.261	-0.0848	0.651	0.670	0.134
Motif 2	-0.109	-0.204	-0.274	0.657	0.636	0.189
Motif 3	-0.236	-0.271	-0.249	0.609	0.600	0.101
Motif 4	-0.325	-0.313	-0.169	0.580	0.582	0.134
Motif 5	-0.266	-0.208	-0.108	0.886	0.897	0.135
Motif 6	-0.406	-0.493	-0.511	0.579	0.107	0.139
Motif 7	-0.536	-0.536	-0.485	0.491	0.0356	0.109
Motif 8	-0.368	-0.330	-0.368	0.312	0.286	0.279

Table S6: Correlation matrix for mRNA levels, translation rates and protein levels of two functionally equivalent genes (i.e., fitness is determined by the sum of two genes' protein levels).

		Gene 1			Gene 2		
		mRNA	Translation	Protein	mRNA	Translation	Protein
Gene 1	mRNA	1	-0.3491	0.5885	-0.2879	-0.3106	-0.5209
	Translation	-0.3491	1	0.5522	-0.3078	-0.2594	-0.4949
	Protein	0.5885	0.5522	1	-0.5217	-0.5002	-0.8904
Gene 2	mRNA	-0.2879	-0.3078	-0.5217	1	-0.341	0.5974
	Translation	-0.3106	-0.2594	-0.5002	-0.3414	1	0.5498
	Protein	-0.5209	-0.4949	-0.8904	0.5974	0.5498	1

Table S7: Transcription-translation correlation and mRNA-protein correlation of genes with different optimal protein levels.

**Gene #**	**Optimal protein level (ln *O*)**	**Correlation coefficient**	
		Transcription-translation	mRNA-protein
1	-5.653	-0.9995	0.06265
2	-4.814	-0.9994	0.1049
3	-2.643	-0.9986	0.003202
4	-1.869	-0.9981	0.06626
5	0.006126	-0.9734	0.1348
6	0.1369	-0.9866	0.08257
7	0.3293	-0.9930	0.09362
8	0.7829	-0.9958	-0.07563
9	0.9896	-0.9971	0.06707
10	1.050	-0.9973	0.09356
11	1.177	-0.9971	0.08134
12	1.581	-0.9980	0.06722
13	1.611	-0.9983	0.01392
14	1.680	-0.9983	0.02384
15	1.788	-0.9982	0.07870
16	1.862	-0.9983	-0.003530
17	2.009	-0.9983	0.01340
18	2.218	-0.9984	0.007559
19	2.379	-0.9986	0.02051
20	3.258	-0.9989	-0.006015

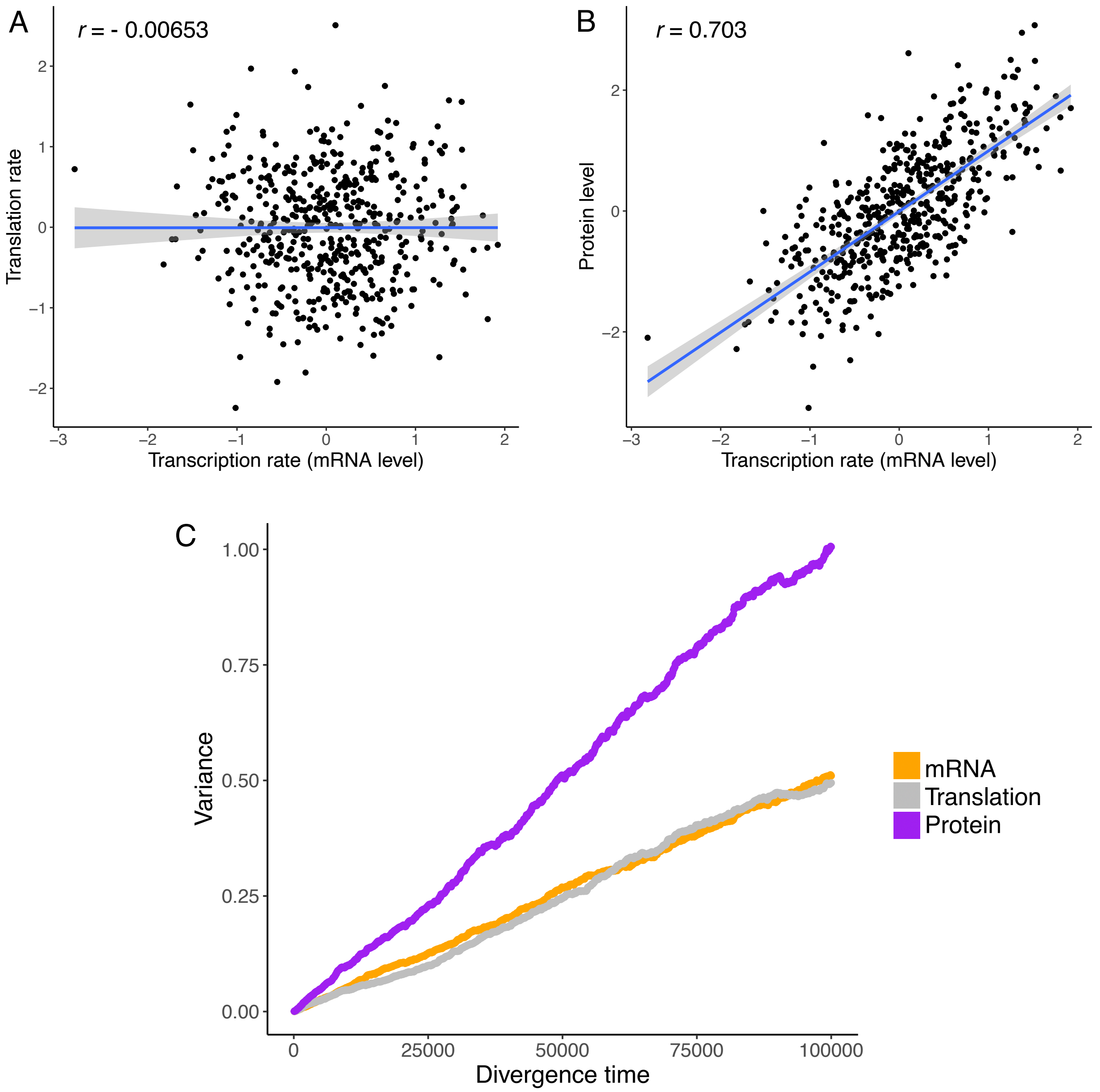

Figure S1: Coevolution of the mRNA level, the rate of translation, and the protein level under neutrality. (A) Variances of the mRNA level, the translation rate, and the protein level through time. (B) End-point correlation between the mRNA level and the translation rate. (C) End-point correlation between the mRNA level and the translation level. Blues lines in (B) and (C) are least-squares regression lines.

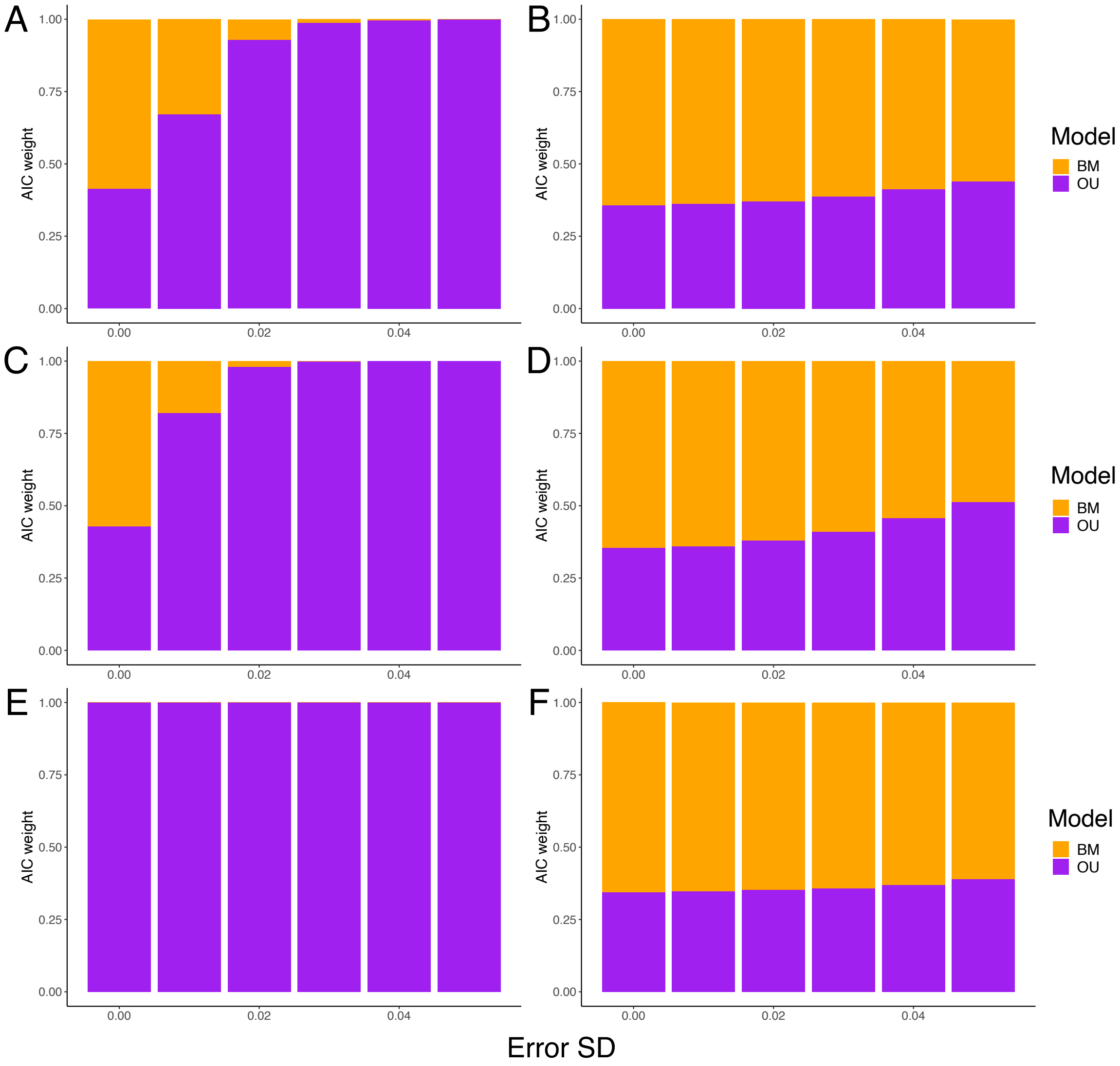

Figure S2: Relative support for Brownian motion (BM) and Ornstein-Uhlenbeck (OU) models when results of simulations along the phylogenetic tree in Fig. 2A, with tip phenotypes subject to different levels of measurement error. For each setting, the extent to which each model is supported is represented by the average AIC weight across 500 independent simulations. (A), (C), and (E) are for results of simulations where the protein level is under stabilizing selection, while (B), (D), and (F) are for simulations of neutral evolution. (A-B) AIC weights computed from the mRNA levels. (C-D) AIC weights computed from the translation rates. (E-F) AIC weights computed from the protein levels.

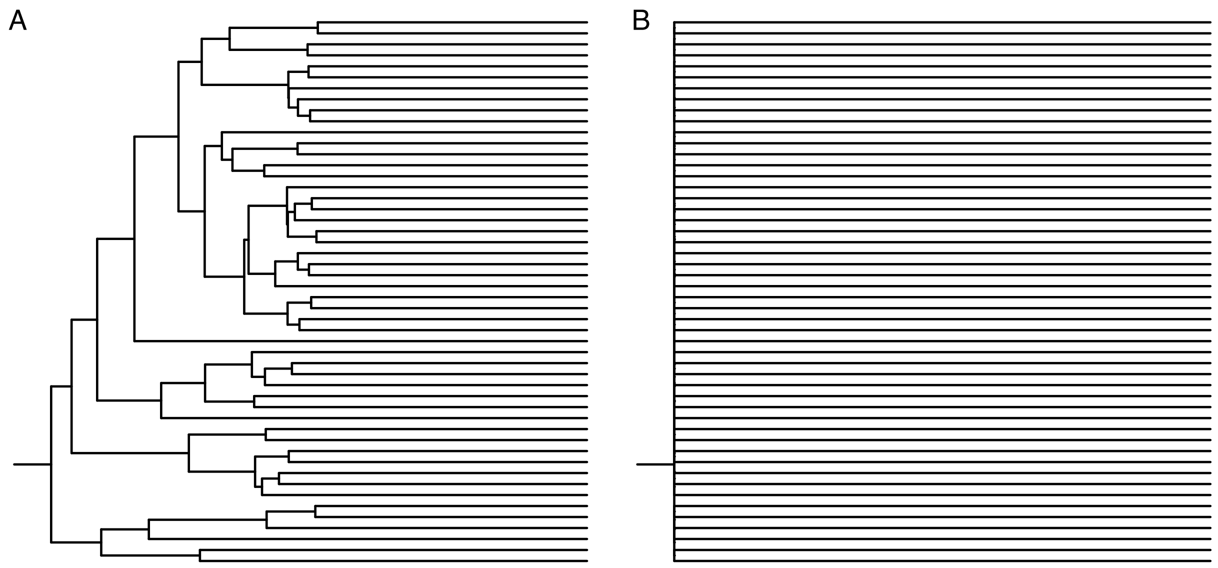

Figure S3: λ-transformed trees used in this study. (A) Tree transformed with $\lambda = 0.5$. (B) Tree transformed with $\lambda = 0$. The root edge is only shown to indicate the root's location. The original tree is shown in Fig. 2A.

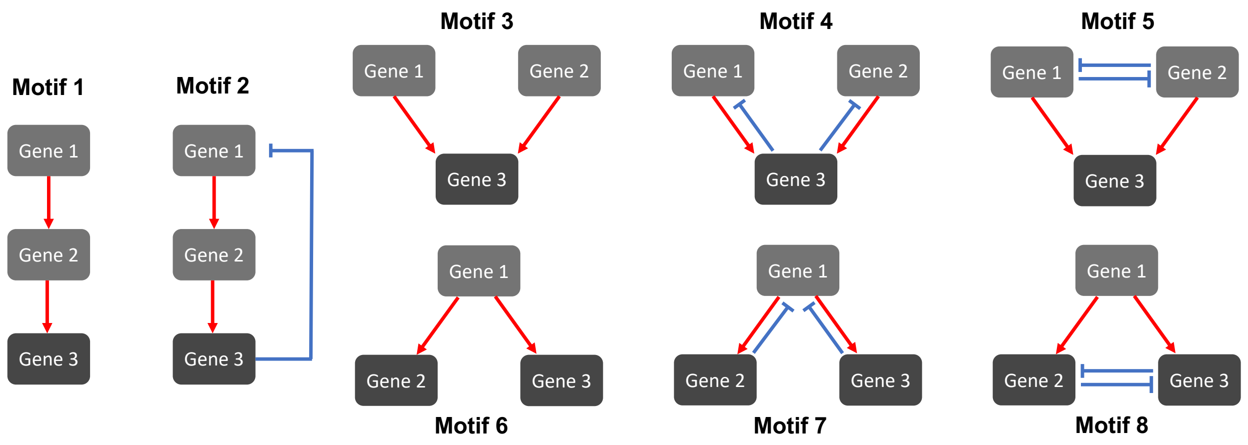

Figure S4: Special cases of triple-gene regulatory network motifs considered in this study. Genes whose protein levels are under direct selection (gene 3 in motifs 1-5, gene 2 and 3 in motifs 6-8) are colored dark gray, while regulators not subject to direct selection are colored light gray. Red arrows represent activation effects ($C_{i,j} = 0.5$), while blue, flat-headed arrows represent repression effects ($C_{i,j} = -0.5$).
